# Bioengineering an Artificial Human Blood–Brain Barrier in Rodents

**DOI:** 10.3390/bioengineering6020038

**Published:** 2019-04-30

**Authors:** Kimia Kamal, Ben Waldau

**Affiliations:** Department of Neurological Surgery, UC Davis Medical Center, Sacramento, CA 95811, USA; kkamal@ucdavis.edu

**Keywords:** blood–brain barrier, organoid, vascularization, perfusion, cerebrovascular disease, immune system, NSG mouse

## Abstract

Our group has recently created a novel in-vivo human brain organoid vascularized with human iPSC-derived endothelial cells. In this review article, we discuss the challenges of creating a perfused human brain organoid model in an immunosuppressed rodent host and discuss potential applications for neurosurgical disease modeling.

## 1. Introduction

Recently, our group has created a human vascularized cerebral organoid model, opening a variety of opportunities to study the human blood–brain barrier in health and disease [1,2]. This review will focus on the mechanism of host-to-graft blood vessel anastomosis, discuss the timing of vascularization, the role of Matrigel in vivo, and applications to study the human blood–brain barrier for neurosurgical disease modeling.

## 2. Bioengineering a Vascularized Human Brain Organoid

Before starting brain organoid vascularization experiments, one of the first questions we asked ourselves was whether the organoid should be vascularized from ‘inside out’ (through injection of endothelial cells into the center of the organoid) or ‘outside in’ (through coating the brain organoids with endothelial cells). To answer this question, we first examined normal embryonic cerebrovascular development as guidance for vascularization strategies. The developing nervous system does not produce endothelial cells [3]. In addition, brain organoids can start their development initially in the absence of blood vessels [4]. In the developing nervous system, blood vessels enter the central nervous system (CNS) from a surrounding perineural vascular plexus. Resident CNS progenitor cells may play a role in the formation of the perineural vascular plexus [5]. There seem to be shared, compartment-specific homeobox proteins that regulate both neural and angiogenic development [6]. Vascularization of the embryonic mouse brain starts on embryonic day 9.5, when vessels grow radially inward from the perineural vascular plexus [7,8]. Therefore, we decided to coat brain organoids with induced pluripotent stem cell (iPSC)-derived human endothelial cells in vitro rather than injecting endothelial cells into the center of the organoid.

**Figure 1:**
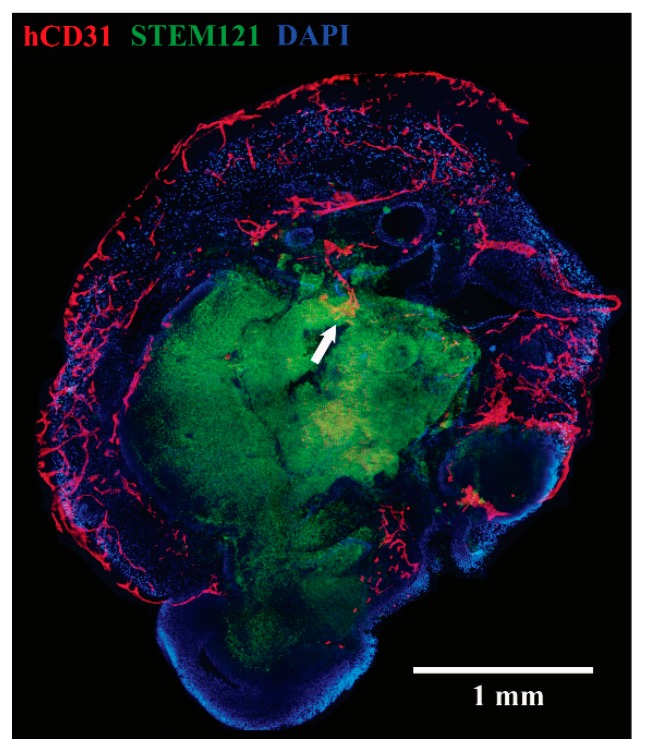
Endothelial cells have penetrated the brain tumor organoid from ‘outside-in’. hCD31 (red), STEM121 (green), DAPI (blue). Reprinted with permission from Pham et al., 2018 [1].

We chose our endothelial cell source based on the results of collaborative disease teams at the Institute for Regenerative Cures at UC Davis. Harding et al. have developed a protocol to differentiate induced pluripotent stem cells into endothelial cells with high purity in eight days without cell sorting [9]. The short period of eight days facilitates the planning of brain organoid vascularization, which we carried out approximately five weeks after organoid-induction. Endothelial cells have self-assembly properties in Matrigel. iPSC-derived endothelial cells were suspended in Matrigel at a concentration of 250,000 cells. On Day 2 after Matrigel embedding, tubule formation can be seen under light microscopy. Two weeks after embedding, vessel sprouts perforated the entire differentiated portion of the organoid (Figure 1). However, our protocol did not result in perfusion of the human endothelial cells with murine blood.

## 3. Bioengineering a Rodent–Human Vascular Anastomosis

Can the human vasculature anastomose with murine endothelial cells and be perfused with mouse blood? Indeed, hybrid human-mouse endothelial cell junctions have been described in the literature. Cheng et al. described a ‘wrapping and tapping’ (WAT) mechanism for the hybrid anastomosis [10]: Human umbilical vein endothelial cells first wrap around host vessels, which have invaded the implant through angiogenesis, and eventually replace them to tap into the blood flow.

**Figure 2:**
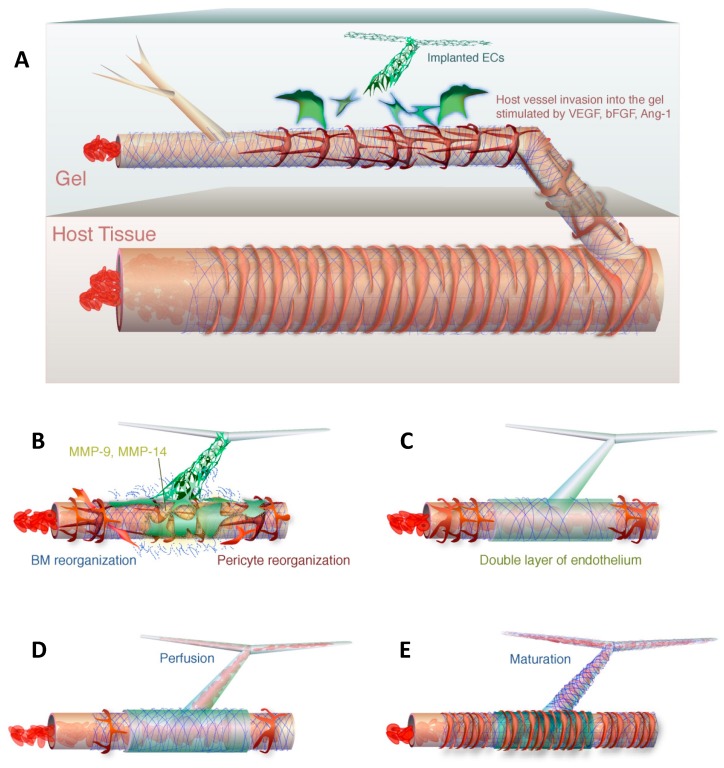
The wrapping-and-tapping mechanism is proposed as a model to explain perfusion of the bioengineered vessel. Host vessels invade a gel with implanted endothelial cells (**A**). The endothelial cells produce metalloproteinases to reorganize the basement membrane (**B**). The engrafted endothelial cells lie on top of the host vessel to form a bilayer (**C**). The host endothelial is opened and blood is allowed to flow into the transplanted endothelial cells (**D**). The perfused segment is fortified with a basement membrane and pericytes (**E**). Reprinted with permission from Cheng et al., 2011 [9].

Figure 2 illustrates such a mechanism. The ‘wrapping and tapping’ mechanism of hybrid vessel anastomosis raises the question of whether our original strategy of pre-coating the brain organoid in vitro before implantation in vivo is the right strategy to achieve perfusion. Surrounding endothelial cells may have no incentive to grow into the organoid since this process is already blocked by vascularization preventing the wrapping and tapping mechanism described above. However, when they first described the WAT mechanism, Munn et al. showed that pre-populating the graft before implantation with endothelial cells does not get in the way of a successful anastomosis. 

One requirement for the wrapping and tapping mechanism is the presence of pericytes, which were not present in our human organoid vascularization experiments. Endothelial tip cells are characterized by their position at the very tip of angiogenic sprouts and by their extensive filopodia protrusions directed towards attractive angiogenic cues [11]. Pericytes stabilize endothelial networks and secrete survival factors [12,13]. Moreover, they induce basement membrane formation along the abluminal surface of the endothelial cell [14,15,16,17]. Pericytes were present not only in the host but also in the graft in Cheng et al.’s hybrid human-mouse endothelial anastomosis experiments leading to vessel perfusion [10]. A successful vessel anastomosis also requires re-arrangement of the basement membrane. Tapping of the basal membrane for hybrid anastomosis requires metalloproteinases 9 and 14 [10]. Metalloproteinase 9 is released by pericytes [18,19]. Metalloproteinase 14 is expressed by smooth muscle cells and other cell types [20]. Both pericytes and smooth muscle cells may therefore be required for successful human organoid perfusion. Vascular endothelial growth factor (VEGF) alone will only result in vascularization but not perfusion of the human brain organoid. 

## 4. Strategies to Achieve Perfusion of Vascularized Brain Organoids

Strategies to achieve perfusion of vascularized human brain organoids will therefore depend on additional factors besides VEGF. Alajati et al. showed that perfusion of human endothelial cells grafted in a matrix can connect to the mouse vasculature in the presence of fibroblast growth factor 2 (FGF-2), platelet-derived growth factor-BB (PDGF-BB), smooth muscle cells (SMCs), or normal human dermal fibroblasts [21]. IPSC-derived endothelial cells in our protocol could thus be supplemented with the original fibroblasts from which they were developed in order to supply the necessary co-factors for perfusion. PDGF-BB regulates pericyte proliferation, motility, and recruitment along the abluminal surface of the endothelial cell [22]. This raises the question from which structure these pericytes will be recruited. The human brain organoid has differentiated into an ectodermal fate while pericytes are derived from the mesenchyme [23]. One possibility is that murine pericytes invade the human endothelial vascular network within the brain organoid. Another possibility is trans-differentiation of iPSC-derived endothelial cells into pericytes or smooth muscle cells. This process has been described in the lung for the transformation of endothelial cells into pericytes in a process called endothelial-mesenchymal transition [24]. An endothelial-to-mesenchymal cell trans-differentiation has also been described for aortic valve endothelial cells into smooth muscle cells [25]. Transforming growth factor β1 (TGFβ1) is important for trans-differentiation of endothelial cells to smooth muscle cells [26,27]. Even mature vascular endothelium has been shown to have the potential for trans-differentiation into smooth muscle cells [28]. Trans-differentiation has also been described between pericytes and fibroblasts [29], which may explain why supplementation with fibroblasts can also lead to vessel maturation and perfusion. Furthermore, trans-differentiation of neural stem cells into smooth muscle cells [30,31] has been described. Considering the potential for trans-differentiation and the necessity of pericytes in both host and graft before vessel anastomosis, it is likely that the process that achieves pericyte coverage of endothelial cells is trans-differentiation, not murine invasion of pericytes. Apart from trans-differentiation, there are techniques described to co-culture iPSC-derived endothelial cells with pericytes [32,33] and early vascular cells [34,35] in order to form vascular networks.

The human blood–brain barrier also relies on astrocytic endfeet which increase its transendothelial electrical resistance [36]. Mature astrocytes are present at 15 weeks gestation in humans [37] and may therefore not be present in the early stages of human brain organoid development. In human cerebral cortical spheroids, astrogenesis was detected between Day 50 and 100, but the astrocytes were not fully mature at this time interval [38]. There may be value in maturing cerebral organoids in vitro for 150 days before vascularization and transplantation into a rodent host to have the optimal concentration of mature astrocytes for competent blood–brain barrier formation.

Once a human vascularized organoid is implanted in the frontal cortex and hippocampus, successful perfusion relies on recruitment of surrounding arterial blood supply. Arterial blood supply in this location is expected to come from multiple sources: The middle cerebral artery, anterior cerebral artery, and posterior cerebral artery via transverse hippocampal artery [39]. Venous drainage may occur via the caudal rhinal vein and transverse hippocampal vein into the dorsal venous system and deep venous system [39]. It is unclear at the present time whether the human endothelial cells will correctly assume an arterial or venous fate after the organoid has already been vascularized. The VEGF gradient seems to play a role in fate determination. Arterial marker genes are expressed in the setting of high VEGF concentration while venous specification occurs in the setting of low and intermediate VEGF concentrations [40]. Human-specific blood–brain barrier evolution could be studied by comparing human vascularized brain organoids, for example, to chimpanzee organoids [41] that have been vascularized.

## 5. The Role of Matrigel in Brain Organoid Engraftment

While Matrigel is important for ingrowth of iPSC-derived endothelial cells into brain organoids in vitro, we found the opposite effect for in vivo engraftment. In our previous work, we transplanted the vascularized organoids after more than two weeks into NSG mice. At that time, Matrigel had mostly been sheared off the organoid in the shaker in order for the surface of the organoid to be in good apposition with the raw surfaces of the resection cavity in the mouse brain after implantation. However, when we supplemented endothelial cells in Matrigel under conditions promoting pericyte ingrowth in order to achieve perfusion, we did not find survival of any organoids three weeks after implantation. The distinction between the two experiments was the time point of organoid re-coating with Matrigel. Organoids did not survive in vivo if they were re-embedded in Matrigel just before transplantation. This finding is in agreement with the observations of our liver organoid team at the Institute of Regenerative Cures. Hepatocytes, for example, survived in vivo for eight weeks after engraftment in a decellularized liver matrix, but only for 3–4 weeks when embedded in Matrigel after splenic or omental injection [42]. Counter-intuitively, Matrigel seems to block in vivo survival of organoid grafts in some circumstances, possibly by creating a barrier between the host brain and the transplant graft. In our experience, an engraftment strategy should be chosen in which the Matrigel has already detached from the organoid surface so that there is direct apposition of the organoid surface with the raw edges of the recipient rodent brain. The process of Matrigel detachment in a shaker takes at least two weeks. Even though endothelial cells and pericytes are initially embedded in the Matrigel, they do not get lost after Matrigel detachment since they have already grown into the organoid after two weeks. Along the same lines, in successful vascularization and perfusion of human liver organoids transplantation experiments, liver organoids were not implanted in Matrigel into the cranial cavity [43]. Instead, iPSC-derived liver buds were detached from the Matrigel and collected before they were used for transplantation. An alternative to Matrigel for successful hepatocyte transplantation has been an inverted colloid crystalloid matrix [44] or endothelial cell-covered hepatocyte spheroid integration [45]. 

## 6. Timing of Vascularization In Vitro

The optimal time point for vascularization of brain organoids in vitro remains to be determined. It cannot be performed too soon after starting the brain organoid protocol since VEGF will disrupt the tight junctions on the organoid surface [46] and possibly lead to non-spherical organoid growth and premature differentiation. VEGF not only increases leakage of the blood–brain barrier [47], it can also decrease non-vascular tight junctions, such as in retinal pigment epithelial cells [48] and hepatocellular cells [49]. We re-coated brain organoids on Day 30 to allow for undisrupted organoid growth for 4 weeks, and organoids continued to grow spherically afterwards despite the presence of VEGF.

## 7. Perfused Human non-Cerebral Organoid Models as a Roadmap to Perfused Human Brain Organoids

Looking at other organoid systems, perfusion of human endothelial cells with murine blood inside the brain organoid should be technically feasible. The perfusion of organoids for different organ systems has been achieved with host-derived vasculature and graft-derived human vasculature. For liver organoids, human endothelial cells connected with their murine counterparts and were perfused within 48 h [43]. Vascularization of kidney organoids has been achieved after subcapsular implantation with host-derived endothelial cells [50]. Kidney organoids have been vascularized with human cells in vitro [51,52]. Vascularization of an intestinal organoid model has been achieved with human umbilical vein endothelial cells (HUVECs) [53]. Likewise, vascularization of embryonic stem cell-derived cardiac muscle was achieved with HUVECs [54]. Currently, perfusion of human brain organoids has only been achieved with murine endothelial cells [55]. The next big step in cerebral organoid research will be perfusion of brain organoids with human endothelial cells to model a human blood–brain barrier.

## 8. Role of the Immune System in the Organoid Blood–Brain Barrier

**Figure 3:**
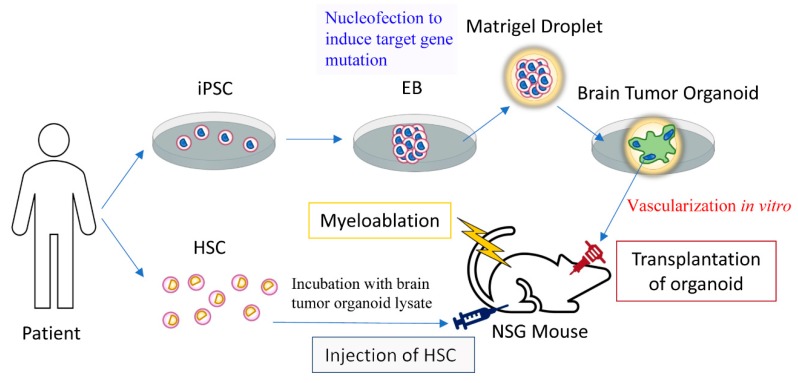
The efficacy of brain tumor immunotherapy could be evaluated in a rodent vascularized human brain tumor organoid model with a humanized immune system. Hematopoietic stem cells (HSC) or their progeny are incubated with brain tumor organoid lysate to prime an immune reaction against the brain tumor organoid. IPSC: Induced pluripotent stem cell, EB: embryoid body.

We have implanted brain organoids into the cranial cavity of non-obese diabetic scid gamma (NSG) mice. NSG mice are non-obese diabetic (NOD)-*scid* mice bearing a mutation in the interleukin-2 gamma chain receptor and lack natural killer cells [56]. The advantage of the NSG mouse model is that it can be engrafted with the human immune system [57,58]. Human immune systems engrafted in the NSG mouse are functional [59,60,61]. Therefore, the criticism that the chimeric NSG mouse-human organoid blood–brain barrier does not take into account the interaction of the immune system in pharmacological testing or disease modeling can be addressed by humanizing the immune system of NSG mice. The immune system may play a vital role when studying the pathology of the blood–brain barrier in a brain organoid disease model. Humanizing NSG mice may be important to study the effect of the immune system on vascularized brain tumor organoids. For brain tumor organoids, activation of the humanized immune system may be the mechanism of tumor control or regression, as the endogenous immune system is important in immunotherapy [62,63]. Figure 3 shows a model of studying immunotherapy of brain tumor organoids with a humanized immune system in an NSG mouse. Brain tumor organoids can be created by introducing oncogenic mutations or oncogene overexpression in brain organoids or by growing a three-dimensional brain tumor cell line [64,65,66]. Adoptive T cell immunotherapy can be studied in the brain tumor organoid model with cytotoxic tumor-specific T cells [67], for example, by priming them with brain tumor organoid lysate before infusion. The efficacy of immunotherapy can be evaluated by screening for involution of a transplanted vascularized brain tumor organoid. Cancer mutations that are recognized by T cells can be identified with whole-exomic sequencing [68] and be validated in such a brain tumor organoid model.

Other neurological diseases that involve the blood–brain barrier and the immune system may be studied with a perfused brain organoid in a mouse with a humanized immune system as well. In a multiple sclerosis model, endogenous Th17 cells were shown to weaken tight junctions at the blood–brain barrier [69]. The blood–brain barrier in Alzheimer’s disease is impaired [70], and the immune system is also thought to play a role [71,72]. A perfused brain organoid may also be used to study the recently discovered glymphatic system [73,74], which may play a role in Alzheimer’s disease.

One potential pitfall of creating a successfully perfused brain organoid model in NSG mice is the altered immunoreactivity of their central nervous system. Resident microglial cells in NSG mice show darker and more intense CD68 immunoreactivity than their WT counterparts [75]. Microglia have been shown to be involved in sealing a leaky blood–brain barrier [76]. Activated microglia modulate the expression of tight junctions of the blood–brain barrier [77] and can lead to its disruption [78]. Therefore, it is unclear whether the microglial environment in NSG mice will be able to create a functional blood–brain barrier.

## 9. Brain Organoids and Organs-On-Chips

There have been significant advances recently in modeling the blood–brain barrier on chips [79]. Organ-on-chips are microfluidic cell culture devices with continuously perfused microchannels inhabited by living cells [80]. Even metabolic coupling of the neurovascular unit can be modeled with an organ-on-a-chip [81]. However, there are important differences between these two models. Currently, vascularized organoids cannot undergo microfluidic perfusion since capillaries are too small to cannulate with a microfluidic pump system. Brain organoids need to be implanted into a host animal model in order to establish perfusion. The blood–brain barrier may be insufficiently modeled since brain organoids are in the very early stages of development. However, they truly model the in situ blood–brain barrier with blood whereas perfusing an organ-on-a-chip with blood may be cumbersome due to activation of the coagulation cascade.

## 10. Neurosurgical Diseases that Could be Modeled with an Artificial Blood–Brain Barrier

**Figure 4:**
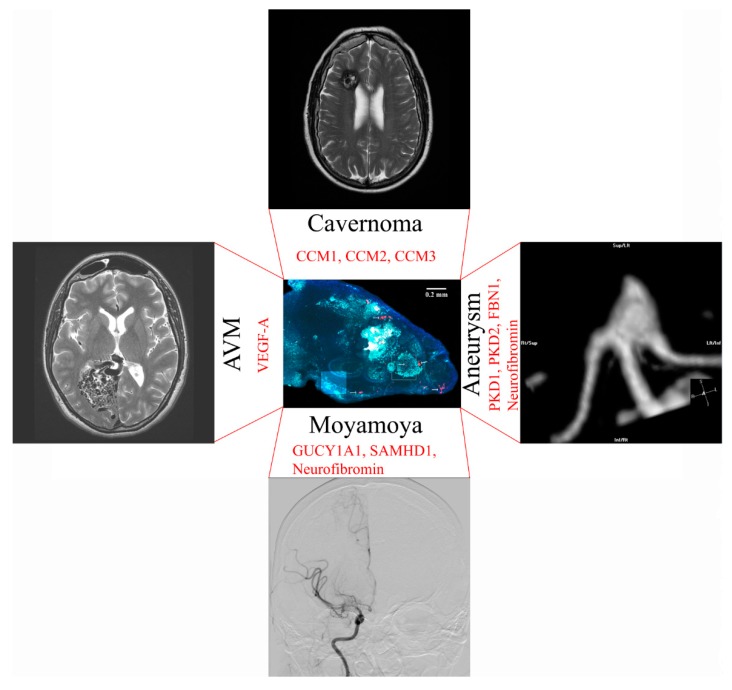
Potential applications to model neurosurgical disease in a vascularized brain organoid with genomic editing. Gene modifications to match the disease phenotype are shown in red.

An artificial human blood–brain barrier model in a rodent could be used to model neurosurgical diseases. An overview of neurosurgical diseases that could be bioengineered with genome editing of vascularized brain organoids is shown in Figure 4. 

## 11. Moyamoya Disease

Moyamoya disease leads to progressive occlusion of skull base arteries, which can result in ischemic stroke or brain hemorrhage [82]. Genetic causes of Moyamoya are sickle cell disease [83,84], neurofibromatosis 1 [85], Noonan syndrome [86], Costello syndrome [87], Alagille syndrome [88], guanylate cyclase 1 soluble subunit alpha 1 (GUCY1A1) mutations [89], SAM domain and HD domain containing protein 1 (SAMHD1) mutations [90], Majewski syndrome [91], Turner syndrome [92], Down syndrome [93], and other causes. Respective mutations could be introduced into iPSC-derived endothelial cells with targeted genomic editing. The contribution of endothelial cells versus brain microenvironment to disease progression could be investigated by introducing mutations concurrently in brain organoids and endothelial cells versus endothelial cells only. Vascularized brain organoids could be transplanted with immunosuppression into a mouse model of sickle cell disease to study disease mechanisms [94]. 

## 12. Cerebral Aneurysm Formation

While most brain aneurysms are acquired, there are also genetic causes of aneurysm formation that could be studied in the human brain organoid vasculature. Genetic causes that have been associated with intracranial aneurysms are polycystic kidney disease [95] and potentially Marfan syndrome with the fibrillin-1 (FBN1) gene [96] and Neurofibromatosis type 1 [97]. Polycystic kidney disease (PKD) is a well-known cause of cerebral aneurysms to neurosurgeons. It can be caused by mutations in PKD1 and PKD2 [98] and may be caused by weakness of smooth muscle cells. Targeted genomic editing could introduce these mutations selectively into iPSC-derived endothelial cells to study mechanisms of PKD development. Acquired aneurysms could be studied with smoke inhalation in rodent vascularized brain organoid models [99]. Smoking is an established risk factor for cerebral aneurysm formation and rupture [100]. 

## 13. Modeling of Arteriovenous Malformations

While the genetic pathogenesis of arteriovenous malformations is largely unknown, AVMs can also be associated with hereditary lesions [101]. Hereditary hemorrhagic telangiectasia, for example, could be studied through modifications in TGFβ signaling in human endothelial cells used for organoid vascularization [102,103,104]. Increased expression of VEGF-A mRNA has been identified in the microarray analysis of AVM tissue [105]. Therefore, overexpression of VEGF-A in the rodent vascularized organoid can potentially be used to study factors contributing to AVM development. A model of human blood vessel organoids has been created [106] which may resemble a model of an AVM after transplantation into the rodent brain.

## 14. Brain Cavernoma Development

Cerebral cavernous malformations can occur sporadically or as a consequence of inherited loss-of-function mutations in the familial cavernoma genes cerebral cavernous malformation 1, 2 and 3 (CCM1, CCM2, and CCM3) [107]. Loss of CCM1, for example, leads to excessive angiogenesis through loss of inhibition of sprouting angiogenesis [108]. The CCM genes could be studied in a knock-down model in human endothelial cells used for vascularization of the organoid. 

## 15. Conclusions

Current in vitro models have significant limitations with respect to modeling neurosurgical cerebrovascular diseases. Perfusion of a human brain organoid may be possible by the tapping and wrapping mechanism once complete blood vessels have been modeled with endothelial cells and pericytes. The influence of the immune system on the blood–brain barrier, such as in a brain tumor organoid model, may be studied by humanizing the immune system of the rodent host. Cerebrovascular diseases, such as Moyamoya disease, AVMs, aneurysms and cavernous malformations could be modeled with genomic editing of endothelial cells used for vascularization.
